# Effective anesthetic management with remimazolam and ketamine without muscle relaxants for parotidectomy in a patient with myotonic dystrophy: A case report

**DOI:** 10.1097/MD.0000000000030415

**Published:** 2022-08-26

**Authors:** Yoshiaki Ishida, Miki (Iwasaki) Habu, Yoshie Toba

**Affiliations:** a Department of Anesthesiology, Seirei Hamamatsu General Hospital, Hamamatsu, Japan.

**Keywords:** bispectral index, flumazenil, ketamine, muscle relaxant, myotonic dystrophy, opioid, remimazolam

## Abstract

Patients with myotonic dystrophy (DM) are highly sensitive to anesthetics, muscle relaxants, and opioids, necessitating appropriate anesthetic management. Recently, remimazolam, an ultra-short-acting benzodiazepine, has been approved for use as a general anesthetic in Japan, and patients with DM have reportedly been treated with remimazolam. However, to the best of our knowledge, no study has reported on endotracheal intubation without the use of muscle relaxants under anesthetic management with remimazolam, nor on the combination of remimazolam and ketamine.

**Patient concerns:**

A 23-year-old man was referred to our hospital for right parotidectomy and diagnosed with DM just before surgery. At the surgeon’s discretion, he was scheduled to undergo nerve monitoring to preserve the facial nerve.

**Diagnosis:**

Myotonic dystrophy.

**Interventions:**

We planned total intravenous anesthesia without muscle relaxants and selected remimazolam for anesthesia. Our aim was to prevent the intraoperative or postoperative complications associated with propofol and inhalational anesthetics. Additionally, we selected multimodal analgesia, including ketamine, to avoid opioid use. General anesthesia was induced with ketamine 30 mg, remifentanil 0.72 μg/kg/min, and remimazolam 12 + 6 mg. Endotracheal intubation was performed under videolaryngoscopy without the use of muscle relaxants. For postoperative analgesia, we administered additional doses of ketamine 20 mg and acetaminophen 1000 mg, and the surgeons infiltrated 8 mL of xylocaine 0.5% with epinephrine into the skin incision before starting the surgery. Intraoperative anesthesia was maintained with remimazolam 0.9 to 1.0 mg/kg/h and remifentanil 0.26 to 0.50 μg/kg/min. Flumazenil was administered for rapid awakening and safe extubation. All vitals, including the bispectral index, were stable during surgery.

**Outcomes:**

The patient did not develop facial nerve paralysis, sore throat, or hoarseness, nor did he have any memory of the surgery. Good postoperative analgesia was achieved.

**Lessons:**

We achieved effective anesthetic management using remimazolam without muscle relaxants in a patient with DM. Furthermore, the combination of remimazolam and ketamine provided good sedation and postoperative analgesia.

## 1. Introduction

Myotonic dystrophy (DM) is an inherited disorder characterized by myotonia and extramuscular features, including cardiac conduction abnormalities and dysphagia.^[1,2]^ The increased sensitivity of patients with DM to anesthetics, muscle relaxants, and opioids may pose serious problems during anesthetic management.^[1]^ Anesthesia-induced postoperative respiratory failure was observed in a patient with DM.^[3]^

Remimazolam, a novel and ultra-short-acting benzodiazepine, was recently approved for use as a general anesthetic in Japan.^[4]^ Studies have reported that remimazolam can be used for the anesthetic management of patients with DM.^[5,6]^ However, to the best of our knowledge, no study has reported endotracheal intubation under anesthetic management with remimazolam without the use of muscle relaxants. Moreover, no study has reported on the combination of remimazolam and ketamine.

Herein, we report effective anesthetic management with remimazolam and ketamine without the use of muscle relaxants for parotidectomy in a patient with DM. The patient provided written consent for the publication of this case report.

## 2. Case presentation

The patient was a 23-year-old man (height, 178.4 cm; weight, 57.8 kg), who was referred to our hospital for right parotidectomy. He was diagnosed with DM just before surgery based on the following episodes and various test results.

He became aware of weakness in grip strength in junior high school and gradually encountered problems in getting up. The family history was notable since his father and uncle had similar symptoms but had not been formally diagnosed with DM. Physical examination revealed distal extremity weakness beyond the wrist joint, grip and percussion myotonia, masseter muscle atrophy, and slight limitation in tongue movements. The serum creatine phosphokinase level was 919 U/L (reference range, 59–248 U/L). Electromyography revealed myotonic discharge. Electrocardiography showed a first-degree atrioventricular block and sinus rhythm at 61 beats per min. There were no abnormal findings on echocardiography or pulmonary function testing.

At the surgeon’s discretion, he was scheduled to undergo nerve monitoring to preserve the facial nerve. Therefore, we suggested the provision of general anesthesia and planned total intravenous anesthesia (TIVA) without muscle relaxants in consultation with the surgeon to ensure the patient’s safety. Remimazolam was selected as the general anesthetic agent. Our aim was to prevent the intraoperative or postoperative complications associated with propofol and inhalational anesthetics. Additionally, we selected multimodal analgesia, including ketamine, to avoid opioid use.

Noninvasive blood pressure monitoring, electrocardiography, pulse oximetry, and bispectral index (BIS) determination were performed intraoperatively. First, we administered ketamine 30 mg and started continuous infusion of remifentanil 0.72 μg/kg/min for analgesia. Thereafter, we administered remimazolam 12 mg intravenously for 1 minute and started continuous infusion at 1 mg/kg/h. Since loss of consciousness (LOC) did not occur after 3 minutes, we also administered remimazolam 6 mg for 1 minute. After confirmation of LOC, we established adequate mask ventilation without the use of muscle relaxants. After laryngeal deployment using a McGRATH^TM^ MAC laryngoscope (Covidien, Medtronic Inc, Tokyo, Japan), we confirmed that the glottis was slightly open, and protectively inserted an 8.0 endotracheal tube into the trachea. Endotracheal intubation was successful, without any incidence of coughing.

Thereafter, we maintained continuous infusion of remifentanil 0.26 to 0.5 μg/kg/min and remimazolam 0.9 to 1.0 mg/kg/h for adequate analgesia and sedation. For postoperative analgesia, we administered ketamine 20 mg and acetaminophen 1000 mg, and the surgeons infiltrated 8 mL of xylocaine 0.5% with epinephrine into the skin incision before starting the surgery. We refrained from using muscle relaxants intraoperatively since the surgery was performed with nerve monitoring to preserve the facial nerve.

**Figure 1.**
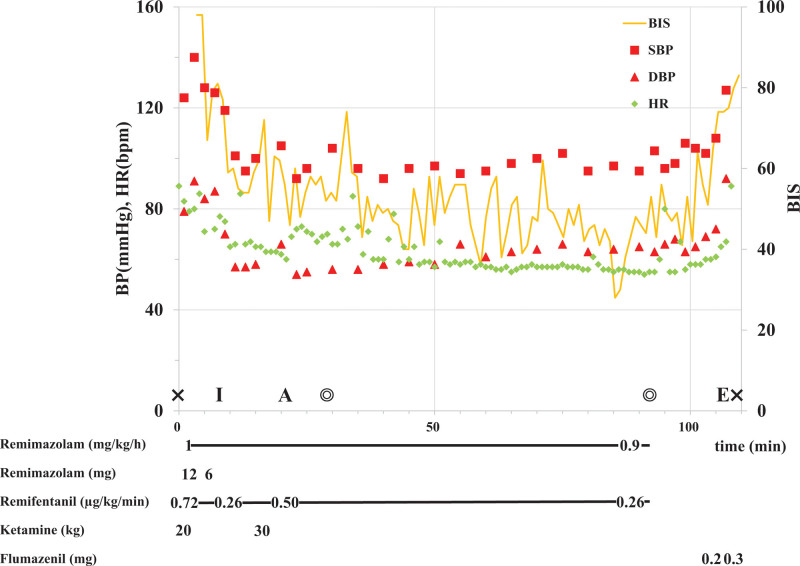
Anesthesia record. Double circles and crosses represent the start/end of surgery and start/end of anesthesia, respectively. A = acetaminophen, BIS = bispectral index, BP = blood pressure, bpm = beats per minute, DBP = diastolic blood pressure, E = extubation, HR = heart rate, I = intubation, SBP = systolic blood pressure.

The duration of surgery was 63 minutes. He started weak spontaneous breathing 10 minutes after termination of remimazolam and remifentanil administration and was administered flumazenil 0.2 mg. When he achieved sufficient tidal volume and could follow instructions, we administered an additional dose of flumazenil 0.3 mg to antagonize the effect of remimazolam and removed the tracheal tube. All vital signs, including the BIS, were stable during surgery (Fig. 1).

A postoperative interview was performed on the day after the procedure. He did not develop facial nerve paralysis, sore throat, or hoarseness, nor did he have any memory of the surgery. He required only a single dose of a nonsteroidal anti-inflammatory drug after surgery.

## 3. Discussion

Regional anesthesia with minimal sedation should be considered whenever possible for patients with DM, and anesthesiologists should be extremely careful whenever general anesthesia is planned.^[1]^ Risk assessment during the perioperative period is especially significant, and appropriate anesthetic management should be initiated, owing to the high sensitivity to anesthetics, muscle relaxants, and opioids.^[1]^

TIVA is commonly used instead of inhalational anesthetics in patients with DM undergoing general anesthesia. Caution must be exercised while administering deep inhalational anesthetics to patients with DM, as it can lead to shivering and a compromised cardiac status.^[1]^ Moreover, inhalation anesthesia with sevoflurane may lead to malignant hyperthermia in patients with DM.^[7]^ In contrast, propofol, which is commonly used for TIVA, may induce a myotonic response,^[8,9]^ prolong recovery after anesthesia,^[10]^ and lead to respiratory depression even at a low concentration.^[2]^ Therefore, we selected remimazolam since our patient had been diagnosed with DM. The efficacy of remimazolam as a sedative-hypnotic for general anesthesia is not inferior to that of propofol, and can stabilize the blood pressure.^[11]^ Doi et al reported that administration of remimazolam 12 mg/kg/h to low-risk surgical patients [American Society of Anesthesiologists-physical status (ASA-PS) 1–2] resulted in a mean time to LOC of 88.7 seconds.^[11]^ Since our patient was young, he did not lose consciousness rapidly and required an additional dose of remimazolam; however, anesthesia was induced without vasopressor administration. Another advantage of remimazolam is the existence of an antagonist, that is, flumazenil. In this case, we effectively achieved adequate depth of anesthesia with remimazolam during surgery and were able to quickly awaken and safely extubate the patient after flumazenil administration.

Muscle relaxants should be avoided as far as possible during administration of general anesthesia in patients with DM, especially if it is not essential.^[1]^ According to previous studies, endotracheal intubation was performed in patients with DM without the use of muscle relaxants.^[8,10]^ However, to the best of our knowledge, endotracheal intubation under anesthetic management with remimazolam without muscle relaxants has not been reported previously. Our primary concerns in this patient were sore throat, hoarseness, and further decline in swallowing function after surgery. Patients often complain of sore throat (27%), hoarseness (27%), and dysphagia (43%) after extubation.^[12]^ Although the use of a videolaryngoscope does not reduce the possibility of sore throat, it improves the glottic view and reduces the possibility of laryngeal injuries, including hoarseness.^[13]^ Moreover, previous studies have not reported any differences in the incidence of sore throat, hoarseness, and laryngeal injuries with or without the use of muscle relaxants.^[14]^ In this case, we achieved good mask ventilation without administering muscle relaxants. Moreover, postoperative sore throat and hoarseness were avoided, as endotracheal intubation was performed protectively using a video laryngoscope. Hence, we achieved successful endotracheal intubation under remimazolam administration without the use of muscle relaxants.

The use of shorter acting opioids is recommended during the intraoperative period for patients with DM, whereas opioids should be avoided during the postoperative period if possible.^[1,15]^ Therefore, we selected multimodal analgesia with ketamine, to avoid opioids as much as possible. However, to the best of our knowledge, no study has reported on the use of ketamine for anesthetic management in conjunction with remimazolam. Ketamine reduce pain by virtue of being a noncompetitive antagonist of the N-methyl-d-aspartic acid receptor, preempting the need for opioid analgesics after surgery.^[16]^ However, ketamine is also a dissociative anesthetic agent that suppresses neuronal function in the cortex and thalamus, and leads to aberrant excitatory activity in the limbic system, including the hippocampus, resulting in electroencephalographic changes distinct from those caused by anesthetics.^[17]^ Ketamine induces a greater increase in the BIS value compared to midazolam in the presence of general anesthesia^[18]^ (ketamine 0.4–0.5 mg/kg increases the BIS value in the presence of propofol or sevoflurane anesthesia^[19–21]^). Moreover, in our patient, we had to particularly refrain from using muscle relaxants intraoperatively since nerve monitoring was needed to preserve the facial nerve, leading to large dose of intraoperative remifentanil. Although a relatively large dose of intraoperative remifentanil may lead to acute opioid tolerance and hyperalgesia,^[22]^ studies have demonstrated that they can be prevented by ketamine^[23,24]^ and withdrawal of remifentanil infusion.^[25,26]^ In this case, even when a total ketamine dose of 0.9 mg/kg was administered during remimazolam infusion, the BIS value was stable and maintained at a range of 40 to 60, which corresponded to the BIS value when low-risk surgical patients (ASA-PS 1–2) were administered remimazolam 0.97 to 0.99 mg/kg/h.^[11]^ We provided multimodal analgesia with ketamine to achieve minimal opioid usage in the perioperative period, leading to good postoperative pain control.

## 4. Conclusions

In summary, we achieved effective anesthetic management using remimazolam without the use of muscle relaxants in a patient with DM. Furthermore, the combination of remimazolam and ketamine provided good sedation and postoperative analgesia. Further studies are needed to assess the feasibility of combining remimazolam and ketamine for this purpose.

## Acknowledgments

We would like to thank Editage (www.editage.com) for the English language editing of this manuscript.

## Author contributions

Conceptualization: Yoshiaki Ishida, Miki (Iwasaki) Habu

Data curation: Yoshiaki Ishida

Supervision: Yoshie Toba

Writing—original draft: Yoshiaki Ishida

Writing—review and editing: Yoshiaki Ishida, Miki (Iwasaki) Habu, and Yoshie Toba
